# Optimization of TMS target engagement: current state and future perspectives

**DOI:** 10.3389/fnins.2025.1517228

**Published:** 2025-01-29

**Authors:** Pantelis Lioumis, Timo Roine, Ida Granö, Dogu Baran Aydogan, Elena Ukharova, Victor H. Souza, Dubravko Kičić, Risto J. Ilmoniemi, Nikos Makris

**Affiliations:** ^1^Department of Neuroscience and Biomedical Engineering, Aalto University School of Science, Espoo, Finland; ^2^BioMag Laboratory, HUS Medical Imaging Center, Aalto University, University of Helsinki and Helsinki University Hospital, Helsinki, Finland; ^3^Cognitive Brain Research Unit, Department of Psychology and Logopedics, Faculty of Medicine, University of Helsinki, Helsinki, Finland; ^4^Advanced Magnetic Imaging Centre, Aalto University, Espoo, Finland; ^5^A.I. Virtanen Institute for Molecular Sciences, University of Eastern Finland, Kuopio, Finland; ^6^Departments of Psychiatry and Neurology, A. Martinos Center for Biomedical Imaging, Center for Morphometric Analysis, Massachusetts General Hospital, Harvard Medical School, Boston, MA, United States; ^7^Department of Anatomy and Neurobiology, Boston University School of Medicine, Boston, MA, United States; ^8^Psychiatric Neuroimaging Laboratory, Harvard Medical School, Brigham and Women’s Hospital, Boston, MA, United States

**Keywords:** TMS–EEG, target engagement, dMRI (diffusion magnetic resonance imaging), structural connectivity, neurophysiological signatures of brain circuits

## Abstract

Neuromodulation is based on the principle that brain stimulation produces plastic changes in cerebral circuitry. Given the intersubject structural and functional variability, neuromodulation has a personalized effect in the brain. Moreover, because of cerebral dominance and interhemispheric functional and structural differences in the same individual, the characterization of specific brain circuitries involved is currently not feasible. This notion is extremely important for neuromodulation treatments applied in neuropsychiatry. Specifically, the efficacy of the neuromodulation treatments is critically dependent on the anatomical precision of the brain target and the circuitry which has been affected by the TMS intervention. Furthermore, for a complete understanding of how the brain behaves under stimulation, the characterization of its neurophysiological response is necessary as well. This goal can be achieved with TMS–EEG guided by current multimodal neuroimaging techniques in real time, namely MRI-based anatomical segmentation and diffusion MRI-based tractographic analysis.

## Introduction

A major contributor to the imperfect response rates using transcranial magnetic stimulation (TMS) is that TMS is not reliably targeted. In current standard-of-care for TMS in gambling disorder (GD), a universal targeting procedure is used based on *x,y,z* MNI coordinates for all subjects. Following this “one target for all” approach, coil positioning and coil orienting leads to poorly defined TMS-induced electric field (E-field) intensity and uncertain target engagement in cortical sites other than the primary motor cortex area (M1). This is a serious challenge, which existing neuroimaging and neurostimulation technologies may be able to solve (Aydogan et al., 2025; Lioumis and Rosanova, 2022; Nieminen et al., 2022; Rosanova et al., 2009).

The localization of cerebral cortical cytoarchitecture is a critical concept and has been emphasized since late 1880s by prominent neuroanatomists such as Brodmann and Vogt (Brodmann, 2005; Vogt and Vogt, 1919), as well as later by Von Economo, Bailey, and Von Bonin (Bailey and Von Bonin, 1957; Economo, 1927), and recently by the neuroimaging community (Human Connectome Project; Glasser et al., 2016; Rushmore et al., 2022; Van Essen et al., 2019; Van Essen et al., 2012; Van Essen and Glasser, 2018). Cortical localization is associated with the structural circuitry of a given area. Areas adjacent to the intended targets may thus be stimulated; these areas may have totally different connectivity patterns. This is related to differences in their cytoarchitectural structure and, most importantly, to their different structural connectivity, which are highly individual (Caspers et al., 2006; Smith et al., 2019). These considerations are key concepts for neuromodulation and, more specifically, for target engagement during TMS. Given that neuromodulation is mechanistically related to Hebbian learning enabled by neuroplasticity, target engagement needs to be defined not only in terms of stimulated cortical area but also, importantly, in terms of brain circuitry associated with that area. Equally important is the characterization of the neurophysiological signature of the stimulated area, which should be combined with the specific underlying structural neuroanatomical background (cortical area and circuitry). Such an approach has not been achieved to date. Neuroimaging allows the identification of visible morphological landmarks; however, the absence of personalized MRI-guided navigation can result in inconsistent and unreliable target definition. Consequently, inaccurate localization of the target results in inconsistent and unreliable target engagement in clinical practice.

## From non-specific stimulation targeting to anatomically and electrophysiologically specified target engagement

A major question raised about TMS interventions is whether the stimulated structural circuitry is specific or not. Current neuroimaging technologies and analysis methods have not been applied efficiently enough to guarantee a precise characterization of cortical targets and their associated circuitry engagement specificity. Cortical anatomical connections are precise and architecturally specific. That is, each cortical field has a specific structural signature in terms of its laminar architecture and the fiber connections related to its different layers (Mesulam, 2000; Pandya et al., 2015; Pandya and Yeterian, 1985). This level of explanation has been understood and elucidated in the non-human primate, such as the macaque (i.e., the rhesus monkey). Nevertheless, in humans, we have not yet been able to determine with certainty the precise and specific cortical structural connectivity beyond the stems of the principal fiber pathways (Makris et al., 2023a,b; Rushmore et al., 2020). Thus, the specific origins and terminations of fiber tracts in the human brain are determined approximately, which provides an incomplete understanding of structural connectivity (e.g., Rushmore et al., 2020). Recent advances in diffusion-based MRI tractography have been hampered by this gap in knowledge of human brain structural connectivity (Makris et al., 2023b; Mesulam et al., 2015; Pandya et al., 2015). Besides that, anatomical understanding, an important factor affecting knowledge of the specific circuitry underlying a cortical area, is hindered by the structural and functional neuroanatomical variability between individuals. Structural and functional variability in humans is a critical factor, especially with personalized medical interventions such as TMS. To minimize these uncertainties, current neuroimaging and neurophysiology techniques can be implemented. More specifically, navigated TMS combined with real-time tractography (Aydogan et al., 2025), a technique that allows the user to see in real time the structural connections of the area under stimulation, helps in detecting the structural cortical and subcortical connectivity matrix of the targeted cortical area to ensure reliability of structural measurements (Makris et al., 2023a,b). On the other hand, TMS combined with fMRI and high-density electroencephalography (EEG) could be implemented to address the functional signature of the stimulated cortical area (Rosanova et al., 2009) and its associated effective connectivity. Following this mapping approach, we can optimize the anatomical, connectional and functional ontologies of a targeted area (Makris et al., 2023a).

## Pre-SMA and SMA: two adjacent cortical areas within Brodmann’s area 6, with different structural circuitries, and different electrophysiological signatures that manifest distinct behavioral/clinical phenomenologies

The pre-supplementary motor area (pre-SMA) structural connectome is very different compared to that of the supplementary motor area (SMA). The pre-SMA is strongly connected with the dorsolateral prefrontal (DLPFC) and anterior cingulate (ACC) cortices. Furthermore, the pre-SMA is connected to the basal ganglia (BG, including the caudate, putamen and subthalamic nucleus (STN)), the thalamus (thal) and the brainstem. Pre-SMA connections with the STN are via the hyperdirect pathway (hd.p), with the brainstem via the medial forebrain bundle (MFB) and with the ACC via the cingulum bundle (CB). Other brain regions such as the nucleus accumbens (NAc) and the subcallosal cortex (SCC) are connected indirectly to the pre-SMA (Barbas and Pandya, 1987; Matelli et al., 1991; Matsuzaka and Tanji, 1996; Nachev et al., 2008; Nachev et al., 2007; Picard and Strick, 1996; Roesch and Olson, 2003; Woolsey et al., 1952; Zilles et al., 1996). Thus, while the pre-SMA is connected principally with anterior areas in the prefrontal and anterior cingulate cortex, the SMA is connected mainly with posterior areas in the parietal and posterior cingulate cortex (Rizzolatti and Luppino, 2001). This notion is of great relevance in TMS therapeutics, in particular in cognitive inhibition. Cortico–subcortical connections are also different between these two cortical regions. The pre-SMA connects strongly with the ventral tegmental area (VTA), which is critical with respect to reward-related inhibition. It also connects with other brainstem areas compared to SMA, which connects with the spinal cord, given its stronger relation with the motor circuitry (Barbas and Pandya, 1987; Matelli et al., 1991; Matsuzaka and Tanji, 1996; Nachev et al., 2008; Nachev et al., 2007; Picard and Strick, 1996; Roesch and Olson, 2003; Woolsey et al., 1952; Zilles et al., 1996).

**Figure 1:**
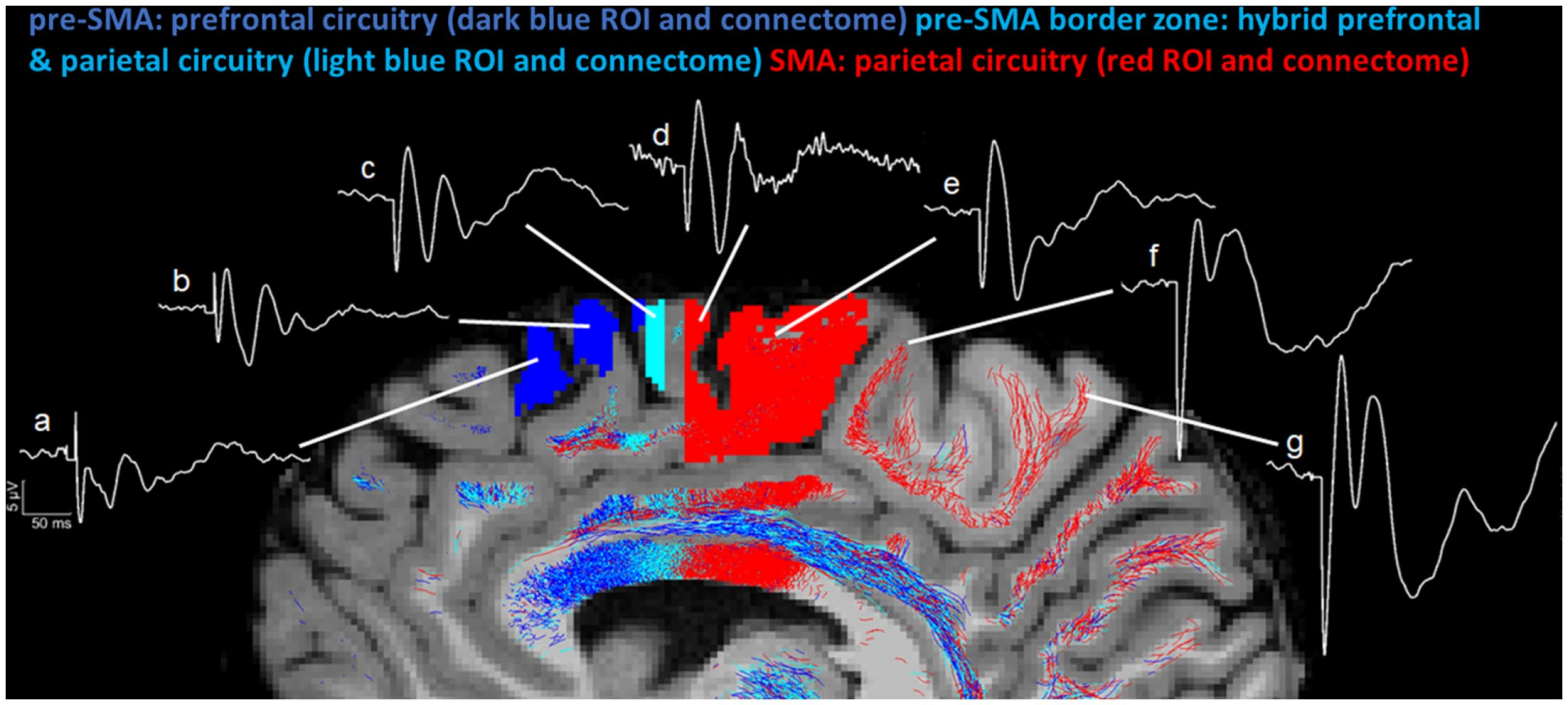
Neurophysiological signatures of distinct anatomical areas based on TMS–EEG evoked responses (TEPs). Averaged TEPs (see Supplementary material for details) have been recorded while stimulating (a) pre-SMA superior, (b) pre-SMA superior posterior (border zone of pre-SMA with SMA), (c) SMA anterior (border zone of SMA with pre-SMA), (d) SMA proper anterior, (e) SMA proper posterior (border zone of SMA with M1), (f) leg premotor cortex, and (g) leg primary motor cortex.

**Figure 2:**
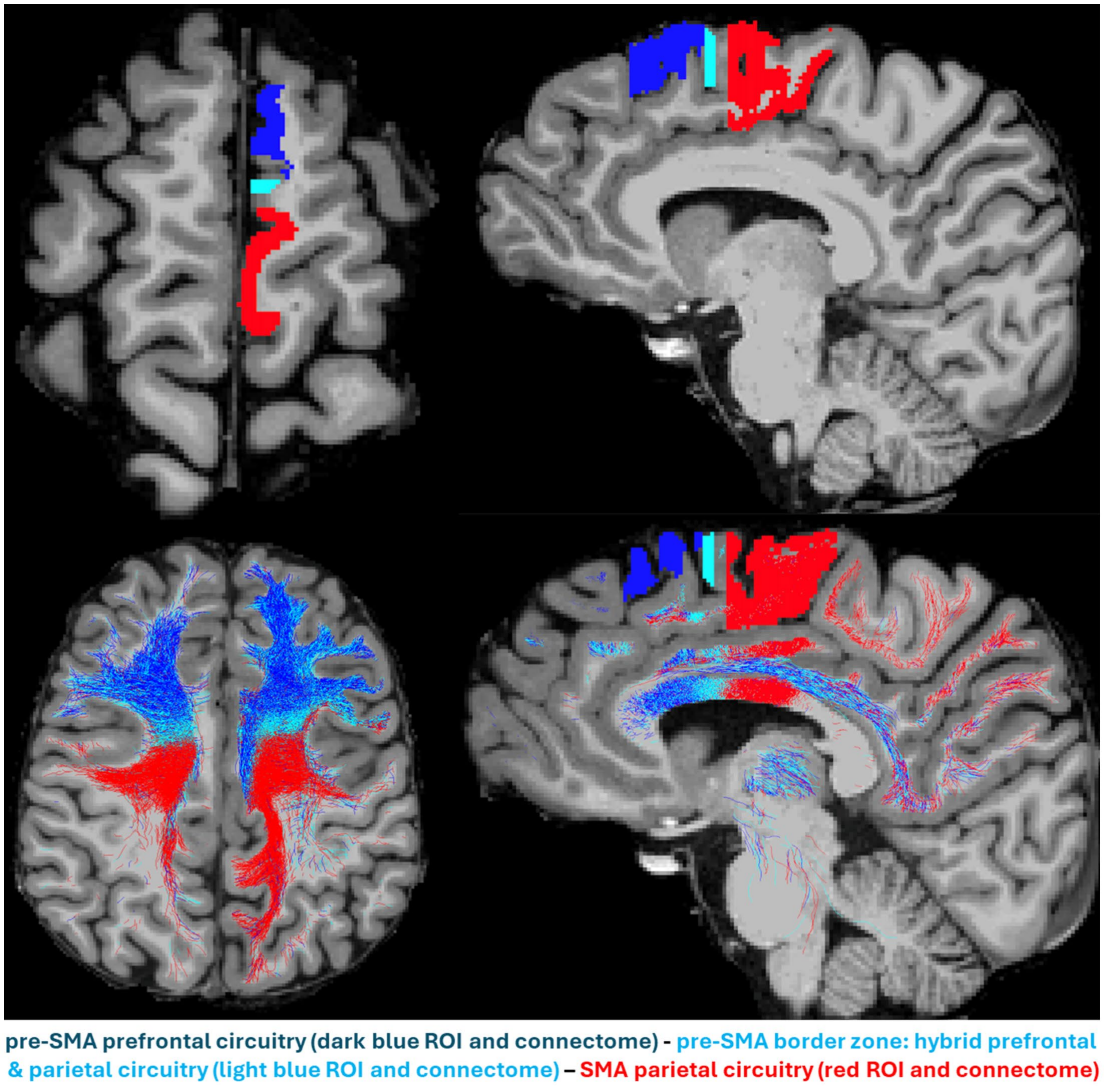
The parallel anterior–posterior gradient in anatomical location and structural circuitry of pre-SMA and supplementary motor area (SMA) in the human brain. The pre-SMA (dark blue), pre-SMA border zone (celestial blue) and SMA (red) are shown in an axial (upper left) and a sagittal (upper right) view. Their structural connectomes as reconstructed by dMRI tractography are shown in an axial (lower left) and a sagittal (lower right) view, following the same color-coding schema, i.e., pre-SMA connectome in dark blue, pre-SMA border zone connectome in celestial blue and SMA connectome in red. The pre-SMA prominent prefrontal circuitry (in blue) located anteriorly, contrasts sharply with the SMA parietal circuitry located posteriorly, whereas the pre-SMA border zone circuitry shows a mixture of both, prefrontal and parietal connections.

Besides the structural and connectional ontological differences of pre-SMA and SMA, we also need to characterize their behavioral and clinical ontologies in clinical settings (Makris et al., 2023a). “Silent” regions in the brain, i.e., areas without observable behavioral responses, deserve special consideration, given that they remind us of the existence of a vast cortical territory of uninterpretable brain function. In part, our inability to determine the role of these areas lies in the technical limitations in clinical behavioral assessment. Another reason could be that our tools cannot elicit and/or detect such activities. In the context of a specific pathology, the behavioral/clinical ontology of cortical targets in neuromodulation could be addressed based on their neurophysiological signatures (Figure 1) and gathered information from their anatomical and structural connectional architectures (Figure 2). For instance, in major depressive disorder (MDD), or more specifically in one of its subtypes, the anhedonic one, it may be of great importance to characterize the specificity of the target. Anhedonic MDD is associated with an alteration of the mesocorticolimbic system, which is represented principally by the medial forebrain bundle (MFB) (Yang et al., 2015; Yang et al., 2014). Targeting the MFB at the cortical level requires diffusion MRI-based tractography mapping to identify the different cortical endings of this fiber system. Given the current limitations of dMRI tractography, the cortical terminations of MFB can be only approximately determined, which could still be used as a potential target area to stimulate subcortical centers associated with this fiber tract. At any rate, based on that information, a strategic decision needs to be formulated in planning target engagement. To this end, more advanced brain stimulation techniques need to be implemented, such as real-time or pre-experiment personalized tractographic targeting and the combined navigated multi-locus TMS (mTMS) with high-density EEG (hd-EEG), which both offer to the user real-time neuroimaging and neurophysiological information, crucial to ensure specificity of the desired target engagement in terms of (a) its accurate cortical location, (b) its specific circuitry signature and, (c) its specific EEG signature. Figure 1 illustrates this concept by means of navigated TMS–EEG (Casarotto et al., 2022; Lioumis and Rosanova, 2022; Rosanova et al., 2009).

As shown in Figure 2, pre-SMA and SMA may need to be stimulated simultaneously to engage prefrontal, parietal and subcortical structures, including larger regions of the thalamus, caudate nucleus, putamen, subthalamic nucleus, the brainstem and the cerebellum. Thus, by combining real-time tractography with results from concurrent mTMS and hd-EEG, we could ensure engagement of adjacent cortical areas (e.g., pre-SMA and SMA) with categorically different cytoarchitectural, structural connectivity, and neurophysiological signatures and thus expect to elicit the whole array of beneficial different behavioral and clinical outcomes.

## Discussion

Technological advancements since the 1950s have allowed us to study the human brain in unprecedented ways. Namely, neuroimaging and neurophysiological techniques enable non-invasive studies of the human brain *in vivo*. More specifically, anatomical T1-, T2- and diffusion-weighted MRI can provide detailed information about cortical structure and its connectivity. Furthermore, the neurophysiological aspect of the cerebrum can be assessed by fMRI and magnetoencephalography (MEG) in a way that we can generate an understanding of a brain function within space and time resolution parameters at the millimeter and millisecond scales of spatial and temporal resolution, respectively. The integration of structural and functional techniques to novel visualizations of neuronal processing has changed dramatically since the late 1990s, especially with the advent of digital brain-image inflation and other 3D rendering techniques of structural brain data (3D Slicer Image Computing Platform, 2024; Dale and Sereno, 1993). The latter approaches can be combined with TMS–EEG, which adds further possibilities in studying brain processes related to “causality” (Ilmoniemi et al., 1997; Massimini et al., 2005), a matter of great importance in science, philosophy, and modern neuroscience. Furthermore, this is crucial in elucidating how brain systems break down by disease and how they recover after treatment. Therefore, an emerging concept based on the above consideration is that the elucidation of processing of brain functions, such as language, memory, and affect, can lead to an understanding on how the brain works as an integrated whole and in segregated networks. Ultimately, the anatomical, connectional, functional, and behavioral/clinical ontologies could be integrated to establish a hub for understanding and elucidating “how the brain works.”

Clinically, this integrative approach seems to be critically relevant with respect to pre-treatment targeting preparation to add specificity, anatomical accuracy and precision of cortical target and circuit target engagement during distinctive therapeutic interventions. Guiding TMS based on anatomical MRI, dMRI tractography, and real-time TMS–EEG mapping enables us to precisely identify a target cortical area, its underlying circuitry and its neurophysiological signature (Rosanova et al., 2009). In practical terms, these neuroimaging and neurophysiological procedures need to be applied prior to TMS intervention to ensure reliable target engagement and the optimal TMS intensity dosage in the individual subject. Moreover, the stimulation parameters, including but not limited to temporal structure, frequency, and targeted electrophysiological state, for each intervention could also be optimized adaptively in closed-loop treatments (Gogulski et al., 2023). Although in principle obvious, this concept has not been clinically practiced to date, albeit both real-time tractography and TMS–EEG can be applied in clinical setups (Edlow et al., 2023; Elhawary et al., 2011) despite the challenges introduced by the complexity of such real-time setups. Generally, what is used in current standard-of-care TMS therapeutics, is a universal targeting procedure based on standard *x*,*y*,*z* MNI or Talairach coordinates or even standard cortical site based on anatomical landmarks, which do not necessarily represent accurately the individual subject’s cortical anatomy. Furthermore, the TMS stimulation procedure outside the motor cortex (e.g., dorsolateral prefrontal cortex) typically entails a specific coil orientation with respect to the morphological correlates of the primary motor cortex (i.e., central sulcus). Consequently, target identification, engaged circuitry, and neurophysiological outcome of the target area can be anatomically inaccurate. Therefore, to engage accurately the desired cortical targets, the above-mentioned neuroimaging procedures need to be performed prior to the therapeutic intervention. These procedures can be ideally performed by the novel mTMS technology (Nieminen et al., 2022) together with the real-time tractography (Aydogan et al., 2025), where neuroimaging-guided automatic mapping of the cortex of unprecedented accuracy (Sinisalo et al., 2024; Tervo et al., 2020, 2022; Matsuda et al., 2024) can be achieved without being affected by user dependence and expertise. Thus, processing of multimodal neuroimaging data could enable the precise anatomical determination of the cortical target and ensure more specific and reliable treatment planning. Conceptually, structural and electrophysiological specificity of cortical targets and circuitry and their reliable engagement is currently feasible following the methodology discussed herein.

## Data Availability

The original contributions presented in the study are included in the article/Supplementary material, further inquiries can be directed to the corresponding author.
