# Supplementary material for: Optimization of TMS target engagement: current state and future perspectives
Source: Front Neurosci. 2025 Jan 29;19:1517228. doi: 10.3389/fnins.2025.1517228 (PMC11814169; doi:10.3389/fnins.2025.1517228)
Supplement: Supplementary file 1 [file Data_Sheet_1.docx]

# Supplementary material

# Methods

One subject (female, 21 years old) participated in the experiment. We first performed the MRI and then, a week after, the TMS–EEG acquisition.

***MRI acquisition***

MRI data were acquired with a Siemens 3T Skyra. Diffusion MRI data were acquired with a state-of-the-art multi-shell sequence with the following parameters: voxel size 2.0 × 2.0 × 2.0 mm^3^, simultaneous multislice (SMS) factor 3, repetition time (TR) 4500 ms, echo time (TE) 133 ms, and 100 gradient orientations distributed in multiple diffusion-weighting shells of 900, 1600, and 2500 s/mm^2^. We acquired twelve images with b=0 s/mm^2^ and three images with reverse-phase encoding for correcting echo planar imaging (EPI) distortions (Andersson et al., 2003).

T1- (with and without fat suppression) and T2-weighted MRI data were acquired with a resolution of 1.0 × 1.0 × 1.0 mm^3^. In addition, three T1-weighted images were acquired with a resolution of 0.8 × 0.8 × 0.8 mm^3^. Fat-suppressed T1 images were used to generate high-quality head models for realistic E-field modelling performed in SimNIBS (Thielscher et al., 2015). The MRI acquisition took one hour.

***T1-weighted image analysis (incl. parcellation)***

The three regions of interest (pre-SMA proper, pre-SMA posterior, and SMA) were segmented manually based on anatomical criteria and visually recognizable landmarks on T1-weighted images. FreeSurfer was used for preprocessing and cortical parcellation of the T1-weighted images (Destrieux et al., 2010; Fischl, 2012). In addition, FIRST, a Bayesian model in FMRIB Software Library (FSL), was used to segment subcortical gray matter structures (Patenaude et al., 2011).

***Diffusion MRI data analysis***

Preprocessing of diffusion MRI data included denoising in MRtrix3 (Veraart et al., 2016) followed by a correction for subject motion (Leemans and Jones, 2009) and distortions caused by eddy currents (Andersson and Sotiropoulos, 2016) and magnetic susceptibility (Andersson et al., 2003) in FSL. B1 field inhomogeneity correction was performed in Advanced Normalization Tools (ANTs) (Tustison et al., 2010; Tax et al., 2022). Statistical parametric mapping was used to rigidly coregister T1-weighted and distortion-corrected diffusion MRI data (Penny et al., 2011).

Structural brain connectivity was obtained from diffusion MRI data with whole-brain tractography performed with constrained spherical deconvolution (CSD) streamlines tractography (Tournier et al., 2007, 2019). With CSD, complex fiber configurations can be reliably estimated and thus, tractography through regions with crossing fibers can be performed (Tournier et al., 2007; Jeurissen et al., 2013). Multi-shell multi-tissue CSD (Jeurissen et al., 2014; Roine et al., 2015; Dhollander et al., 2019) was used to reconstruct 100 million streamlines by seeding from the white matter–gray matter interface using the probabilistic 2^nd^-order integration (iFOD2) algorithm with default fiber tracking parameters in MRtrix3 (Tournier et al., 2007, 2019). Anatomically constrained tractography was used to improve the anatomical feasibility of the streamlines (i.e., that they begin from gray matter and pass through white matter until gray matter or the spinal cord is reached) (Smith et al., 2012). Segmentation of tissue types used for seeding and anatomically-constrained tractography was based on the corregistered T1-weighted images (Fischl et al., 2004; Patenaude et al., 2011). Regions of interest defined based on the corregistered T1-weighted images were used to extract structural connections specific to them from the whole-brain tractogram.

***TMS–EEG acquisition***

The subject was seated in a comfortable chair and was instructed to relax and fixate on a black cross 3 meters away during recordings. The experiment was carried out with a Nexstim neuronavigated TMS system (NBT 5.0, Nexstim Plc., Finland) with a 70-mm-radius cooled figure-of-eight coil and individual T1-weighted magnetic resonance images. The EEG signals were recorded with a BrainAMP EEG system (Brain Products GmbH, Germany) with a 62-channel Easycap with passive Ag/AgCl-sintered electrodes. The EEG signals were low-pass filtered at 1,000 Hz and sampled at 5,000 Hz.

The EEG electrodes were prepared by scraping the skin under each electrode with an abrasive paste (OneStep AbrasivPlus, H + H Medical Devices, Germany), after which each electrode was filled with a conductive gel (Electro-Gel, ECI, Netherlands), to keep the impedances below 5 kΩ throughout the experiment. The ground and reference electrodes were placed on the right zygomatic bone and mastoid, respectively. Electromyography was recorded with Nexstim EMG; the electrodes were placed in a belly-tendon montage on the right abductor pollicis brevis (APB) muscle.

During the recordings, active noise masking consisting of white noise with mixed-in click sounds (Russo et al., 2022; <https://github.com/iTCf/TAAC>) was played to the subject through in-ear earphones (ER3C Insert Earphones, Etymotic Research Inc., United States). The noise level was adjusted before the experiment until the subject was unable to hear the click at 80% maximum stimulator output (MSO) with the coil held 10 cm above the vertex. The absence of auditory evoked potentials was confirmed by visual inspection of average TEPs across 30 trials.

The APB hotspot was identified as the coil location and orientation that produced maximal and consistent motor-evoked potentials (MEPs). The resting motor threshold (rMT) was estimated with Nexstim’s built-in algorithm (Awiszus, 2003). The rMT was 61% MSO.

A total of seven recordings along the midline were performed (pre-SMA to M1) according to the parcellations. First, the most anterior target was mapped for a coil location, orientation and intensity that produced artefact-free TEPs with early peak-to-peak amplitudes (< 50 ms) of 6–10 µV based on averaging 20 TEPs. The resulting stimulation intensity was 71% MSO. Next, for each target in the parcellation (see Fig. 1), a good coil location was determined based on the same criteria. After the selection of each target, 100 pulses were recorded from that cortical site. The same coil orientation (lateral-medial) and intensity were used for all targets.

***TMS–EEG analysis***

The TMS–EEG data were pre-processed in MATLAB R2024a with a script based on eeglab2021.0 (Delorme and Makeig, 2004) and the TESA package (Rogasch et al., 2017). Epochs were generated by selecting −1.5…1.5 s around each TMS pulse, after which baseline correction was applied (baseline period: −100…−5 ms) by subtracting the baseline average from the signals. The TMS pulse artefact was removed by cutting and interpolating −2…6 ms around the TMS pulse with a cubic interpolation. Channels and trials were visually inspected, and those with high noise were removed (one channel and 8 trials per recording were removed on average). Then, independent component analysis was applied to remove ocular artefacts from the data. Another baseline correction was applied, and the SOUND (lambda = 0.01) and SSP–SIR algorithms (0–10 ms) (Mutanen et al., 2016, 2018) were utilized to remove remaining noise and muscle artefacts. A bandpass filter from 0 to 200 Hz and a notch filter from 48 to 52 Hz were applied to remove high-frequency and line noise. Finally, the terminals were snipped to −1…1 s around the TMS pulse. For each recording, the channel with the largest range in the time window 15…50 ms after the TMS pulse was chosen for visualization (pre-SMA recordings: F1; SMA recordings: FC1; premotor and M1: CP1).

From the TEPs, we extracted their deflection peak amplitudes and latencies as shown in Table 1, and the dominant frequencies. The dominant frequencies were calculated following the approach of (Rosanova et al., 2009). For each channel, event-related spectral perturbation (ERSP) was calculated with EEGLAB (Delorme and Makeig, 2004), i.e., the power spectrum was calculated (window span: 3 to 0.8 wavelet cycles) over all trials, after which the trials were averaged and normalized by subtracting the mean baseline. A bootstrap approach was utilized to consider only significant activation with respect to the baseline (alpha = 0.01). The global ERSP was calculated by averaging the ERSPs over all channels. To estimate the dominant frequency, the ERSP was summed over the time interval 20…100 ms, after which the frequency with the highest power was selected within the 8…50 Hz interval.

| Target/  peaks | I | II | III | IV | V | VI | VII | Dominant frequency |
| --- | --- | --- | --- | --- | --- | --- | --- | --- |
| a | –6.5 µV  12 ms | –0.9 µV  19 ms | –5 µV  44 ms | 0.5 µV  58 ms | –3.2 µV  82 ms | –1.8 µV  93 ms | 1.7 µV  156 ms | 35 Hz |
| b | –3.1 µV  11 ms | 4.5 µV  20 ms | –5.4 µV  43 ms | 2.8 µV  60 ms | –2.1 µV  82 ms | –0.1 µV  93 ms | 0.8 µV  150 ms | 34 Hz |
| c | –7.4 µV  10 ms | 5.3 µV  21 ms | –6.3 µV  42 ms | 2.5 µV  58 ms | –3.9 µV  82 ms | –2.3 µV  94 ms | 3.0 µV  155 ms | 35 Hz |
| d | –6.7 µV  10 ms | 6.8 µV  21 ms | –9.0 µV  43 ms | 4.4 µV  62 ms | –2.8 µV  102 ms |  | 3.0 µV  143 ms | 29 Hz |
| e | –7.9 µV  9 ms | 6.9 ms  23 ms | –9.2 µV  45 ms | –2.8 µV  68 ms | –3.7 µV  78 ms | –1.4 µV  94 ms | 2.9 µV  150 ms | 43 Hz |
| f | –18 µV  9 ms | 6.4 µV  28 ms | 0.1 µV  41 ms | 5.3 µV  68 ms | –7.1  120 ms |  |  | 39 Hz |
| g | –18 µV  10 ms | 12.4 µV  27 ms | –3.1 µV  43 ms | 8.3 µV  61 ms | –12.1  104 ms |  | –1.2 µV  154 ms | 16 Hz |

**Table 1.** *Peak amplitudes and latencies of all the averaged TEPs from each target, and also the dominant frequency at which each cortical site was perturbed (see below Fig. 1).*

Andersson, J. L. R., Skare, S., and Ashburner, J. (2003). How to correct susceptibility distortions in spin-echo echo-planar images: application to diffusion tensor imaging. *Neuroimage* 20, 870–888. doi: 10.1016/S1053-8119(03)00336-7

Andersson, J. L. R., and Sotiropoulos, S. N. (2016). An integrated approach to correction for off-resonance effects and subject movement in diffusion MR imaging. *Neuroimage* 125, 1063–1078. doi: 10.1016/j.neuroimage.2015.10.019

Awiszus, F. (2003). TMS and threshold hunting. *Suppl Clin Neurophysiol* 56, 13–23. doi: 10.1016/s1567-424x(09)70205-3

Delorme, A., and Makeig, S. (2004). EEGLAB: an open source toolbox for analysis of single-trial EEG dynamics including independent component analysis. *Journal of Neuroscience Methods* 134, 9–21. doi: 10.1016/j.jneumeth.2003.10.009

DESTRIEUX, C., FISCHL, B., DALE, A., and HALGREN, E. (2010). Automatic parcellation of human cortical gyri and sulci using standard anatomical nomenclature. *Neuroimage* 53, 1–15. doi: 10.1016/j.neuroimage.2010.06.010

Dhollander, T., Mito, R., Raffelt, D., and Connelly, A. (2019). Improved white matter response function estimation for 3-tissue constrained spherical deconvolution.

Fischl, B. (2012). FreeSurfer. *Neuroimage* 62, 774–781. doi: 10.1016/j.neuroimage.2012.01.021

Fischl, B., Salat, D. H., van der Kouwe, A. J. W., Makris, N., Ségonne, F., Quinn, B. T., et al. (2004). Sequence-independent segmentation of magnetic resonance images. *Neuroimage* 23 Suppl 1, S69-84. doi: 10.1016/j.neuroimage.2004.07.016

Jeurissen, B., Leemans, A., Tournier, J.-D., Jones, D. K., and Sijbers, J. (2013). Investigating the prevalence of complex fiber configurations in white matter tissue with diffusion magnetic resonance imaging. *Hum Brain Mapp* 34, 2747–2766. doi: 10.1002/hbm.22099

Jeurissen, B., Tournier, J.-D., Dhollander, T., Connelly, A., and Sijbers, J. (2014). Multi-tissue constrained spherical deconvolution for improved analysis of multi-shell diffusion MRI data. *Neuroimage* 103, 411–426. doi: 10.1016/j.neuroimage.2014.07.061

Leemans, A., and Jones, D. K. (2009). The B-matrix must be rotated when correcting for subject motion in DTI data. *Magn Reson Med* 61, 1336–1349. doi: 10.1002/mrm.21890

Mutanen, T. P., Kukkonen, M., Nieminen, J. O., Stenroos, M., Sarvas, J., and Ilmoniemi, R. J. (2016). Recovering TMS-evoked EEG responses masked by muscle artifacts. *NeuroImage* 139, 157–166. doi: 10.1016/j.neuroimage.2016.05.028

Mutanen, T. P., Metsomaa, J., Liljander, S., and Ilmoniemi, R. J. (2018). Automatic and robust noise suppression in EEG and MEG: The SOUND algorithm. *NeuroImage* 166, 135–151. doi: 10.1016/j.neuroimage.2017.10.021

Patenaude, B., Smith, S. M., Kennedy, D. N., and Jenkinson, M. (2011). A Bayesian model of shape and appearance for subcortical brain segmentation. *Neuroimage* 56, 907–922. doi: 10.1016/j.neuroimage.2011.02.046

Penny, W. D., Friston, K. J., Ashburner, J. T., Kiebel, S. J., and Nichols, T. E. (2011). *Statistical Parametric Mapping: The Analysis of Functional Brain Images*. Elsevier.

Rogasch, N. C., Sullivan, C., Thomson, R. H., Rose, N. S., Bailey, N. W., Fitzgerald, P. B., et al. (2017). Analysing concurrent transcranial magnetic stimulation and electroencephalographic data: A review and introduction to the open-source TESA software. *NeuroImage* 147, 934–951. doi: 10.1016/j.neuroimage.2016.10.031

Roine, T., Jeurissen, B., Perrone, D., Aelterman, J., Philips, W., Leemans, A., et al. (2015). Informed constrained spherical deconvolution (iCSD). *Med Image Anal* 24, 269–281. doi: 10.1016/j.media.2015.01.001

Rosanova, M., Casali, A., Bellina, V., Resta, F., Mariotti, M., and Massimini, M. (2009). Natural frequencies of human corticothalamic circuits. *J. Neurosci.* 29, 7679–7685. doi: 10.1523/JNEUROSCI.0445-09.2009

Russo, S., Sarasso, S., Puglisi, G. E., Dal Palù, D., Pigorini, A., Casarotto, S., et al. (2022). TAAC - TMS Adaptable Auditory Control: A universal tool to mask TMS clicks. *Journal of Neuroscience Methods* 370, 109491. doi: 10.1016/j.jneumeth.2022.109491

Smith, H. J., Pettigrew, T. F., Pippin, G. M., and Bialosiewicz, S. (2012). Relative deprivation: a theoretical and meta-analytic review. *Pers Soc Psychol Rev* 16, 203–232. doi: 10.1177/1088868311430825

Tax, C. M. W., Bastiani, M., Veraart, J., Garyfallidis, E., and Okan Irfanoglu, M. (2022). What’s new and what’s next in diffusion MRI preprocessing. *NeuroImage* 249, 118830. doi: 10.1016/j.neuroimage.2021.118830

Thielscher, A., Antunes, A., and Saturnino, G. B. (2015). Field modeling for transcranial magnetic stimulation: A useful tool to understand the physiological effects of TMS? *Annu Int Conf IEEE Eng Med Biol Soc* 2015, 222–225. doi: 10.1109/EMBC.2015.7318340

Tournier, J.-D., Calamante, F., and Connelly, A. (2007). Robust determination of the fibre orientation distribution in diffusion MRI: non-negativity constrained super-resolved spherical deconvolution. *Neuroimage* 35, 1459–1472. doi: 10.1016/j.neuroimage.2007.02.016

Tournier, J.-D., Smith, R., Raffelt, D., Tabbara, R., Dhollander, T., Pietsch, M., et al. (2019). MRtrix3: A fast, flexible and open software framework for medical image processing and visualisation. *Neuroimage* 202, 116137. doi: 10.1016/j.neuroimage.2019.116137

Tustison, N. J., Avants, B. B., Cook, P. A., Zheng, Y., Egan, A., Yushkevich, P. A., et al. (2010). N4ITK: improved N3 bias correction. *IEEE Trans Med Imaging* 29, 1310–1320. doi: 10.1109/TMI.2010.2046908

Veraart, J., Novikov, D. S., Christiaens, D., Ades-Aron, B., Sijbers, J., and Fieremans, E. (2016). Denoising of diffusion MRI using random matrix theory. *Neuroimage* 142, 394–406. doi: 10.1016/j.neuroimage.2016.08.016
